# Enhancing Evidence-Based Practice Competence and Professional Skills Using Infographics as a Pedagogical Strategy in Health Science Students: Insights from the InfoHealth Project

**DOI:** 10.3390/ejihpe14040060

**Published:** 2024-04-02

**Authors:** Eva-María Navarrete-Muñoz, Desirée Valera-Gran, Jonatan García-Campos, Carlos Lozano-Quijada, Sergio Hernández-Sánchez

**Affiliations:** 1 Grupo de Investigación en Terapia Ocupacional (InTeO), Department of Surgery and Pathology, Miguel Hernández University, 03550 Alicante, Spain; enavarrete@umh.es; 2 Institute for Health and Biomedical Research (ISABIAL-FISABIO Foundation), 03010 Alicante, Spain; jgarcia@umh.es; 3 Department of Behavioural Sciences and Health, Miguel Hernández University, 03550 Alicante, Spain; 4 Center for Translational Research in Physiotherapy, Department of Surgery and Pathology, Miguel Hernández University, 03550 Alicante, Spain; clozano@umh.es (C.L.-Q.); sehesa@umh.es (S.H.-S.)

**Keywords:** infographics, health sciences, professional development, evidence-based practice, communication skills

## Abstract

Infographics have been recognised as effective visual tools for concise and accessible communication of data and information in various higher education disciplines, particularly in medical education. However, there is a lack of research on their impact on professional skills, difficulty levels, motivation, and overall satisfaction in health science students. Thus, the present study aimed to analyse the knowledge and usage of graphical resources among health science students and evaluate their competencies, the level of challenge they encountered, and their motivation and satisfaction after completing an infographic creation activity. The InfoHealth educational innovation project, conducted by five teachers from the Faculty of Medicine, served as the context for this study, with 143 students voluntarily participating. The intervention involved students working in groups of 2–3 and selecting their own topics for the infographics while receiving instruction, online guidance, and feedback from teachers. A questionnaire was administered to assess students’ perception of skill development and satisfaction with the activity. The findings revealed that schemes were the most recognized and used graphical summaries, compared to synoptic tables and Venn diagrams. The activity led to an increase in professional skills acquisition, motivation, and satisfaction, positively impacting students’ interest in evidence-based practice and reinforcing their knowledge. This study highlights the potential of infographics as a pedagogical tool for competency development and student engagement in health science education, suggesting the need for further research comparing infographics with traditional study methods and involving multidisciplinary teams to promote essential skills for future clinical practice.

## 1. Introduction

Infographics are visual representations that enable the concise and comprehensible communication of data and information [1]. Combining graphics, images, and text in the same communicative element has become an excellent method to communicate complex concepts in an accessible and even entertaining way [2,3], providing additional value to written information [4,5].

From an educational perspective, the use of infographics has shown its efficacy as a useful pedagogical tool in higher education teaching [6,7,8,9,10,11]. Previous studies have emphasised infographics as a pedagogical tool in the teaching of languages [12], mathematics [13,14], science [6], and technology [15], among other disciplines. In particular, the use of infographics as an active learning strategy is spreading throughout medical education [3,11,16]. From a pedagogical point of view, the use of visual communication tools offers an important support system for the cognitive processes involved in solving complex problems, boosting motivation during learning [17] and reducing the cognitive load of students [18]. In this sense, infographics can be a relevant visual resource for the teaching–learning process since they harmonise technological skills with creativity, scientific information search, critical appraisal, and information synthesis [7,8,9,19,20]. Likewise, student participation in creating content that is accessible and of public interest increases their motivation [20]. The responsibility of creating materials for informative purposes is another key element of the use of infographics as a teaching tool [19], which makes it a resource of successful value, especially in the training of students in health specialities.

In scientific dissemination, the use of infographics is an added value in the presentation of the results of a scientific article [16,21,22,23,24,25]. In fact, an increasing number of scientific journals have incorporated the graphical summary as another element of the manuscript [26]. Prestigious journals such as the *British Medical Journal* have a specific section on infographics (https://www.bmj.com/infographics) for the graphic presentation of the key elements of a scientific article. This new editorial initiative of the current scientific publication is supported by recent research that has examined the use and potential of this tool in terms of the dissemination of scientific evidence. The study by Huang et al. showed that scientific articles that were disseminated with infographics on social networks had a higher number of visits and downloads compared to those that did not have this graphical resource [22,27]. In addition, another positive value of the infographics of scientific articles is that they are shared up to eight times more on social networks compared to manuscript abstracts in text format [21].

Despite the considerable educational value that infographics possess as tools for teaching and learning, no studies have been conducted to determine the potential impact they can have on the skills, level of difficulty, motivation, and overall satisfaction of Spanish students pursuing health science degrees such as physiotherapy, occupational therapy, and podiatry. Another important point is to determine the extent to which students enrolled in these degree programs are familiar with and use graphical summaries like infographics as study aids. Thus, the educational innovation initiative InfoHealth was launched. This project involved creating and designing an infographic involving students of physiotherapy, occupational therapy, and podiatry at Miguel Hernández University. In this study, we analysed the preceding knowledge and usage of graphical resources among students who partook in the InfoHealth project. We also evaluated their competencies, the level of challenge they encountered, their motivation, and their overall satisfaction after completing the task.

## 2. Materials and Methods

### 2.1. Design and Description of the Learning Strategy Innovation

The InfoHealth educational innovation project was proposed by five teachers from the Faculty of Medicine at Miguel Hernández University (MHU). This project aimed to improve the learning experience of students enrolled in physiotherapy, podiatry, and occupational therapy degrees. The teachers implemented an activity within various core subjects during the second semester of the 2019/2020 academic year. This activity involved creating an infographic within the following subjects: general procedures in physiotherapy III and physiotherapy in clinical specialities I (for the physiotherapy degree), sports podiatry (for the podiatry degree), and occupational therapy in childhood and adolescence (for the occupational therapy degree).

Prior to creating the infographics, each teacher held a classroom session with their respective students to outline the objectives of the activity, the expected final product, and the significance of the activity’s contribution to the continuous assessment of the subjects involved, which accounts for 10% of the final grade. During this session, the teachers also presented the 12 essential tips for creating effective infographics, as developed by the project team [28]. In addition, the session also covered some basic concepts on how to use infographic design tools, including online tools like Canva^®^, to help students create their infographics. The 12 tips shared with the students included: (1) identifying the target audience; (2) establishing the infographic’s purpose; (3) crafting an engaging title for the target audience; (4) selecting clear and concise information; (5) effectively connecting graphical elements to tell a story; (6) emphasising the main ideas; (7) creating an initial draft of the infographic; (8) following basic principles of graphic design; (9) selecting appropriate colours; (10) testing the infographic to ensure optimal performance; (11) checking for possible typos; and (12) developing an efficient dissemination plan. In addition, in this session, teachers highlighted the importance of using evidence-based health information to create the infographic. Therefore, students were urged to document the content they included in the infographic from suitable scientific references or through other reliable sources.

The infographics creation was a group activity, with each group comprising 2–3 students. The groups had the freedom to choose the theme of their infographic as long as it related to the contents covered in their respective subject. As part of the project requirements, each group was also tasked with preparing a comprehensive written document, including the title of the infographic, personal information of all group members, and a thorough explanation of the developmental process undertaken. This document also had to contain a description of the information search and selection strategies, initial ideas, sketches, and the final proposal. Examples of infographics created by students in each bachelor’s degree program are available in the Supplementary Materials (File S1).

Each group scheduled at least two tutorial sessions with each teacher to ensure quality and progress. Teachers conducted the online tutorials because of the COVID-19 pandemic and the state of emergency. In these virtual sessions, they offered guidance and valuable feedback regarding the topic’s development. They oversaw the information search and selection process, while also providing practical help to the groups in the design and development of their infographics.

### 2.2. Participants

At the beginning of the course, students were informed verbally and in writing about the activity of creating infographics through the university noticeboard. The sample for this study was collected by convenience sampling, in which 143 students partook out of a possible 206. The study did not involve potential risks to the participants, and their participation was voluntary. Therefore, the study did not require approval from an ethical committee but only required participant permission, which was obtained through informed consent. By ensuring informed consent and voluntary participation, ethical considerations were addressed, promoting respect for autonomy and protecting the rights and well-being of the participants. We also ensured full compliance with the Spanish Organic Law 3/2018, of 5 December, on the Protection of Personal Data and the guarantee of digital rights. This law aligns with the principles outlined in the Declaration of Helsinki and further safeguards participant privacy.

### 2.3. Assessment of the Experience

As part of the InfoHealth project, students were asked to complete a questionnaire after the activity. The questionnaire was designed using Google Forms, following the CHERRIES checklist for internet-based surveys [29]. The questionnaire comprised four sections:

(1) Academic and personal information: this section collected basic demographic data about the participants.
(2) Prior knowledge and use of graphical summaries: this section assessed students’ prior knowledge of infographics and their experience using them in educational settings.
(3) Perception of skill development after creating infographics: This section focused on student perceptions of their skill development after participating in the infographic creation activity. The questionnaire listed specific competencies targeted in the activity, which were extracted from the core curriculum requirements for the subjects in which the activity was conducted. Students rated their perceived improvement in each competency using a Likert scale. The internal consistency of this section was assessed using Cronbach’s alpha, which yielded a score of 0.86, indicating good reliability.
(4) Evaluation of satisfaction with the activity: this section assessed student satisfaction with the infographic creation activity.

The questionnaire took approximately 10–15 min to complete.

### 2.4. Statistical Analysis

Statistical analysis was performed with the statistical software R version 4.2.1 (R Foundation for Statistical Computing). All statistical tests were bilateral, with a significance level of 5%. Quantitative variables were described by mean and standard deviation, and qualitative or categorical variables were described by “n” and percentage. The mean and standard deviation in each group were used to compare competence acquisition and the assessment of the activity according to prior knowledge and the use of infographics. We estimated *p* values using the Student statistical *t*-test.

## 3. Results

Of the 143 participants in the InfoHealth project, 58.5% were male with a mean age of 22.3 years (standard deviation 5.3). Around two-thirds (67.8%) of the participants were physiotherapy undergraduates, 7.7% had another previous degree, and 86.7% were second-year undergraduates (Table 1).

Table 2 displays the percentage of students who had previous knowledge or had used graphical summaries. Overall, the results of the initial evaluation performed prior to the activity showed that the percentage of students with prior knowledge of graphical summaries was higher than the percentage of students who used these resources.

Regarding the type of graphical summary, we observed a greater difference between knowledge and use for timelines (54.5%), concept maps (48.2%), flowcharts (41.3%), and idea maps (39.1%). In terms of knowledge, outlines (96.5%) and concept maps (94.4%) were the most familiar resources, while synoptic tables (9.8%) and Venn diagrams (16.8%) were the least familiar. As for usage, less than half of the participants reported using any graphical summaries overall, except for diagrams, which were reported by 86.7% of the participants. The most used resource was concept maps (46.2%) and the least used was Venn diagrams (0%). Likewise, 65.7% were familiar with infographics before starting the activity; this percentage was slightly higher in physiotherapy students (82.5%) and much lower in occupational therapy students (19.4%). We observed no differences in the knowledge of infographics according to sex, course, or previous qualification. Regarding previous use of infographics, 39.9% reported having used them, although we found large differences according to degree (54.6% in physiotherapy, 8.3% in occupational therapy, and 10.0% in podiatry), course (42.7% in second year, 7.7% in third year, and 50.0% in fourth year), sex (49.2% in men and 33.7% in women), and previous degree (39.4% in those with no previous degree; 45.5% in those who had a previous degree).

Table 3 shows the results of the competencies gained after the infographic creation activity. On a scale from zero (not at all) to 10 (very much), the competence with the highest acquisition level after completing the infographic was “I am able to establish good interpersonal communication to address efficiently and empathetically the community where I work and the individuals with whom I interact”, with an average score of 8.54. The competence with the lowest acquisition level was “I am able to formulate hypotheses, evaluate information and promote viable solutions to professional cases and situations”, with an average score of 7.66. When considering prior knowledge or use of infographics, clear differences were observed for all competencies. Students who had knowledge or had used infographics prior to the activity showed a higher acquisition level overall (Table 3).

The students’ assessment of the infographics activity, both for the total and distinguishing by prior knowledge and use of infographics, is shown in Table 4. The students showed, on a scale of 0 to 10, that the difficulty level was moderate (mean of 6.20 for search and sketch, and mean of 6.24 for the entire process). In addition, the students reported that the motivation for the activity was high (mean of 8.08) and that they were highly satisfied overall with the activity and with the level of learning (mean of 8.95 and 8.81, respectively). Regarding the level of satisfaction with the teaching staff, materials, and classmates, working with classmates was the best-valued aspect (mean of 4.50) on a scale from 0 to 5. No differences were observed in the difficulty level according to previous knowledge or use of infographics. In contrast, greater motivation and satisfaction were observed in the students who knew or had previously used infographics, except for their assessment of the materials provided. Over 90% of the students declared they would recommend or partake in this activity again, and they reported agreeing that this activity reinforced their interest in evidence-based practice and their knowledge gained in the degree.

## 4. Discussion

The results of the InfoHealth project have shown that a high percentage of students had knowledge about different graphical summaries before carrying out the infographics activity, compared to students who reported using these graphical resources. Regarding the type of graphical summary, the biggest differences between knowing and using them were found in timelines, concept maps, flowcharts, and idea maps. Diagrams were the most familiar, as opposed to synoptic tables and Venn diagrams, which were the least familiar. Before conducting the activity, infographics were graphic summary techniques known by six out of 10 students and used by four out of 10 students, with slight differences being observed according to gender, degree, previous degree, or course. Once the infographics activity was completed, students showed a high level of competence acquisition, with the results being slightly higher in those students who knew or had previously used infographics. In addition, we observed a moderate level of difficulty and a high level of motivation and satisfaction with the activity. All students involved would recommend or partake in the activity again and were convinced that this activity can increase their interest in evidence-based practice and reinforce their knowledge gained in the degree.

Our study uncovered a notable disparity between the number of students who had previous knowledge of graphical summaries and those who reported using them as learning tools. However, the lack of similar studies in the existing literature prevented us from comparing these results to previous findings. In the absence of evidence, several potential reasons could explain the lower usage of these resources. Earlier research reported that factors such as the extra time required for their use, lack of teacher encouragement, students’ inability to recognise the impact of their use on exam results, or resistance to change might contribute to a lower use of graphical tools [30,31]. Our study also revealed that the most frequently employed graphical summaries were outlines, concept maps, and infographics. The literature has documented that these visual resources can enhance retention and facilitate metacognitive improvements [32,33,34,35]. Conversely, Venn diagrams and synoptic tables were the least used graphical summary tools. This discrepancy could be attributed to the fact that students may be familiar with these tools, but cannot recognise them by their names. For example, while Venn diagrams are very common in mathematics and logic, students may not be aware of this term.

The competence that received the highest rating following the activity was “I am able to establish good interpersonal communication to address efficiently and empathetically the community where I work and the individuals with whom I interact”. This result is in line with previous studies reporting that infographics appear to be a powerful dissemination tool [16,21,22,23,24,25]. According to some recent studies, infographics may play a vital role in connecting with communities and contributing to health literacy [36,37]. Hence, introducing this learning resource to physiotherapy, occupational therapy, and podiatry students could be a pedagogical element of interest for its contribution to improving students’ health communication skills. In contrast, the skills of formulating hypotheses and critical appraisal using the scientific method were the skills least gained by making infographics, according to the students in this study. Despite the need to synthesise information and critically evaluate the scientific literature to create an infographic, graphical representation does not seem the best resource for acquiring specific scientific competencies. However, in this study, we observed that students who had knowledge or had used graphical summaries prior to the activity scored higher in the acquisition of all the competencies assessed. This might be because students who had greater knowledge and/or skills in this graphical representation were more inclined towards the activity. Perhaps, their familiarity with the resource could have influenced a more accurate evaluation of skill acquisition through the activity.

Another interesting result of this study is that the students showed a high level of motivation and satisfaction with the infographics, which coincides with what has been described in previous experiences in nursing [38] or other health sciences [8]. According to our students, the activity played a significant role in increasing their interest in evidence-based practice, while also strengthening their knowledge gained during their degree. This finding is consistent with the results of a study conducted by Saunders et al. [39] in Scotland, where it was observed that the grade on an infographic assignment closely mirrored the grade on the subsequent exam. Taken together, these results suggest that engaging students in activities that encourage evidence-based practice can yield a favourable impact on their interest and comprehension of the subject matter. Moreover, our students reported a moderate level of difficulty in completing the activity, with no significant differences observed between those with or without prior knowledge, or between those who had or had not used infographics before. However, it should be noted that the project team that provided 12 recommendations for the creation of infographics may have played a role in reducing the perceived difficulty of the task. In addition, the use of web applications that offer predefined templates for creating infographics, along with their intuitive nature, may have further contributed to the students’ perceived ease of completing the task.

While this study provides valuable insights into using infographics as a learning tool, it is important to consider some limitations that may affect the interpretation of the findings. First, the level of skills among the students before the activity was not measured, so we did not have information about their starting point. Second, the absence of a comparison group prevents us from conclusively attributing the observed results solely to participation in the InfoHealth project, as other external factors may have influenced the outcomes. It is also important to acknowledge that the voluntary nature of the activity meant that only the most motivated students partook, which could introduce a potential selection bias. Moreover, the questionnaire, while assessing student perceptions, did not capture detailed self-reflection on their learning experiences, limiting our understanding of how students perceived their own skill development during infographic creation. Finally, physiotherapy students were over-represented compared to students from occupational therapy or podiatry programs.

Despite these limitations, this study has several strengths. First, students were from three different health science degrees, allowing for diverse perspectives and experiences. Second, the project facilitated interactions and relationships among the teaching staff involved, promoting collaboration and cross-disciplinary learning. Third, the pre-established recommendations for creating infographics [28] provided a standardised approach for the students. Finally, a comprehensive evaluation of the activity was conducted, including assessing competencies, difficulty, motivation, and satisfaction. This thorough evaluation provides valuable information for the design and implementation of future initiatives.

## 5. Conclusions

The InfoHealth project provides valuable insights into the use and knowledge of graphical summaries among students of physiotherapy, occupational therapy, and podiatry at MHU. The results showed a significant lack of knowledge about graphical summaries and limited use of study tools among university students. However, creating infographics as a pedagogical strategy may be a promising tool for developing competencies and student engagement. Notably, the level of difficulty perceived by students was moderate, mainly when recommendations on creating infographics were provided.

Our findings provide a valuable foundation for understanding the impact of infographics in Spanish health science programs. Future research efforts could build upon our work by systematically investigating whether creating infographics significantly changes student learning outcomes compared to traditional study routines. This could involve employing rigorous research designs, such as randomised controlled trials or pre-test/post-test designs, tailored to measure specific learning objectives relevant to the health science disciplines studied. For example, future research could investigate the impact of infographics on knowledge retention through long-term follow-up assessments, or assess the development of critical thinking skills by analysing student responses to open-ended questions requiring application of the learned material. In addition, investigations into the benefits of multidisciplinary collaboration during infographic development are warranted. Such collaborations could involve partnerships between healthcare professionals, graphic designers, and educational specialists. These collaborations could be further explored to assess the impact on faculty development programs designed to support innovative teaching methods, including the integration of infographics into health science curricula. Overall, the InfoHealth project has shown the potential of infographics as a pedagogical tool for promoting competencies and engagement among students. The findings of this study provide a groundwork for future research aimed at optimising the use of infographics as a teaching and learning tool across various health disciplines.

## Figures and Tables

**Table 1:** General characteristics of students partaking in the InfoHealth project (n = 143).

Variables	% (n)
Sex	
Men	58.5 (83)
Women	41.5 (59)
Age, mean (SD)	22.3
Bachelor’s degree	
Physiotherapy	67.8 (97)
Occupational therapy	25.2 (36)
Podiatry	7.0 (10)
Previous bachelor’s degree	
No	92.3 (132)
Yes	7.7 (11)
Year of study	
Second year	86.7 (124)
Third year	9.1 (13)
Fourth year	4.2 (6)

Abbreviations: SD, standard deviation.

**Table 2:** Percentage of students who had previous knowledge or had used graphical summaries (n = 143).

Graphical Summaries	Knowledge	Usage
	% (n)	% (n)
Timelines	96.5 (138)	86.7 (124)
Concept maps	94.4 (135)	46.2 (66)
Outlines	68.5 (98)	14.0 (20)
Idea maps	60.8 (87)	21.7 (31)
Comparative charts	58.7 (84)	32.9 (47)
Flowcharts	44.1 (63)	2.8 (4)
Organisational charts	28.7 (41)	4.2 (6)
Sequence diagram	23.1 (33)	9.8 (14)
Venn diagram	16.8 (24)	0.0 (0)
Synoptic tables	9.8 (14)	4.2 (6)

**Table 3:** Mean (standard deviation) of the level of skills acquisition on a scale from 0 to 10 after completing an infographic activity based on previous knowledge and usage of infographics (n = 143).

Competencies	Total	Previous Knowledge			Previous Usage		
		No (n = 49)	Yes (n = 94)	*p* Value *	No (n = 86)	Yes (n = 57)	*p* Value *
I am able to formulate hypotheses, evaluate information, and promote viable solutions to professional cases and situations.	7.66 (1.22)	7.08 (1.19)	7.97 (1.21)	**<0.001**	7.26 (1.18)	8.28 (1.00)	**<0.001**
I am able to analyse, evaluate, and assess individual and collective situations, identify problems, interpret data, and formulate solutions to individual or collective problems.	7.72 (1.29)	7.10 (1.48)	8.04 (1.06)	**<0.001**	7.34 (1.33)	8.30 (1.00)	**<0.001**
I am able to appraise information critically and apply the scientific method to improve professional practice.	7.67 (1.33)	6.90 (1.10)	8.07 (1.26)	**<0.001**	7.17 (1.21)	8.42 (1.15)	**<0.001**
I am able to establish good interpersonal communication to address efficiently and empathetically the community where I work and the individuals with whom I interact.	8.54 (1.31)	8.12 (1.52)	8.76 (1.13)	**0.012**	8.31 (1.42)	8.88 (1.05)	**0.011**
I am able to update, consolidate, and integrate new knowledge to improve my professional practice using continuous self-learning techniques and critical analysis.	8.13 (1.18)	7.61 (1.24)	8.39 (1.06)	**<0.001**	7.85 (1.20)	8.54 (1.02)	**<0.001**
I am able to use and promote innovation and creativity to solve professional problems.	7.85 (1.37)	7.37 (1.48)	8.10 (1.24)	**0.002**	7.55 (1.35)	8.30 (1.27)	**0.001**

* *p*-value calculated using the Student *t*-test. Bold values indicate statistically significant *p*-values (*p* < 0.05), suggesting strong evidence against the null hypothesis and indicating an unlikely occurrence by chance alone.

**Table 4:** Assessment of the infographic activity based on previous knowledge and usage of infographics (n = 143).

Competencies	Total	Previous Knowledge			Previous Usage		
		No (n = 49)	Yes (n = 94)	*p* Value *	No (n = 86)	Yes (n = 57)	*p* Value *
Level of difficulty in searching information and drafting (range scale from 0 to 10), mean (SD).	6.20 (1.47)	6.22 (1.61)	6.18 (1.41)	0.867	6.16 (1.45)	6.25 (1.53)	0.743
Level of difficulty in making the infographic (range scale from 0 to 10), mean (SD).	6.24 (1.57)	6.47 (1.78)	6.12 (1.44)	0.204	6.23 (1.57)	6.25 (1.58)	0.961
Level of motivation for making the infographic (range scale from 0 to 10), mean (SD).	8.08 (1.59)	7.57 (1.61)	8.35 (1.51)	**0.005**	7.69 (1.64)	8.68 (1.30)	**<0.001**
Level of overall satisfaction with the work completed, and the result achieved (range scale from 0 to 10), mean (SD).	8.95 (1.15)	8.61 (1.29)	9.13 (1.04)	**0.011**	8.61 (1.24)	9.46 (0.78)	**<0.001**
Level of learning from the chosen topic for making the infographic (range scale from 0 to 10), mean (SD).	4.32 (0.77)	4.08 (0.81)	4.45 (0.73)	**0.007**	4.16 (0.79)	4.56 (0.68)	**0.002**
Level of satisfaction with the teachers (range scale from 0 to 5), mean (SD).	4.05 (0.86)	3.96 (0.91)	4.10 (0.83)	0.369	4.01 (0.86)	4.11 (0.86)	0.525
Level of satisfaction with the instructional materials (range scale from 0 to 5), mean (SD).	4.50 (0.89)	4.33 (1.01)	4.59 (0.81)	0.098	4.36 (1.01)	4.70 (0.63)	**0.014**
Students who would recommend this activity to other classmates, % (n).	98.6 (141)	100.0 (49)	97.2 (92)	-	98.8 (85)	98.2 (56)	-
Students who would partake in the activity again, % (n).	97.9 (140)	100.0 (49)	96.8 (91)	-	97.7 (84)	98.2 (56)	-
Students who think that this type of activity will increase their interest in evidence-based practice, % (n).	97.9 (140)	93.9 (46)	100.0 (94)	-	96.5 (83)	100.0 (57)	-
Students who think that this type of activity reinforces knowledge gained in the degree, % (n).	99.3 (142)	98.0 (48)	100.0 (94)	-	98.8 (85)	100.0 (57)	-

* *p*-value calculated using the Student *t*-test. Abbreviations: %, percentage; SD, standard deviation. Bold values indicate statistically significant *p*-values (*p* < 0.05), suggesting strong evidence against the null hypothesis and indicating an unlikely occurrence by chance alone.

## Data Availability

The data that support the findings of this study are available from the corresponding author [D.V.-G.] upon reasonable request.

## Supplementary Materials

The following supporting information can be downloaded at: https://www.mdpi.com/article/10.3390/ejihpe14040060/s1, File S1: Examples of infographics.
