# Supplementary material for: Enhancing Evidence-Based Practice Competence and Professional Skills Using Infographics as a Pedagogical Strategy in Health Science Students: Insights from the InfoHealth Project
Source: Eur J Investig Health Psychol Educ. 2024 Apr 2;14(4):929–40. doi: 10.3390/ejihpe14040060 (PMC11049272; doi:10.3390/ejihpe14040060)

File S1. Examples of Physiotherapy students.

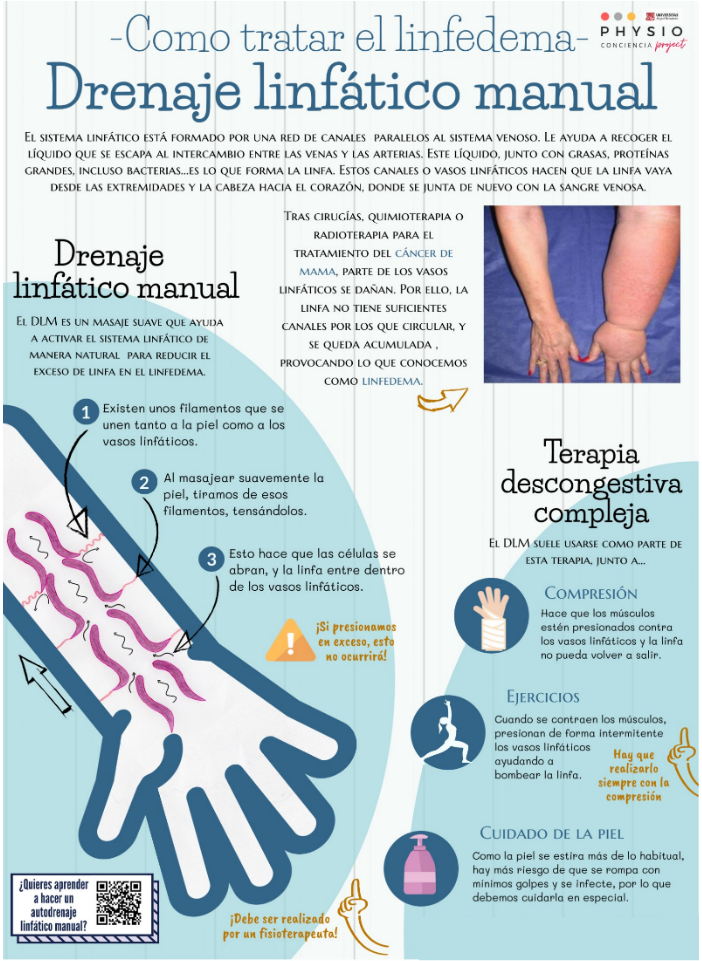

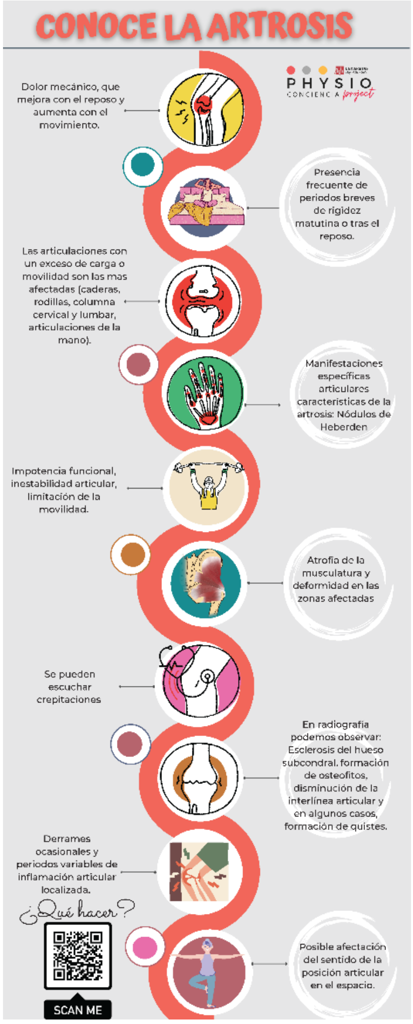

Examples of Occupational Therapy students.

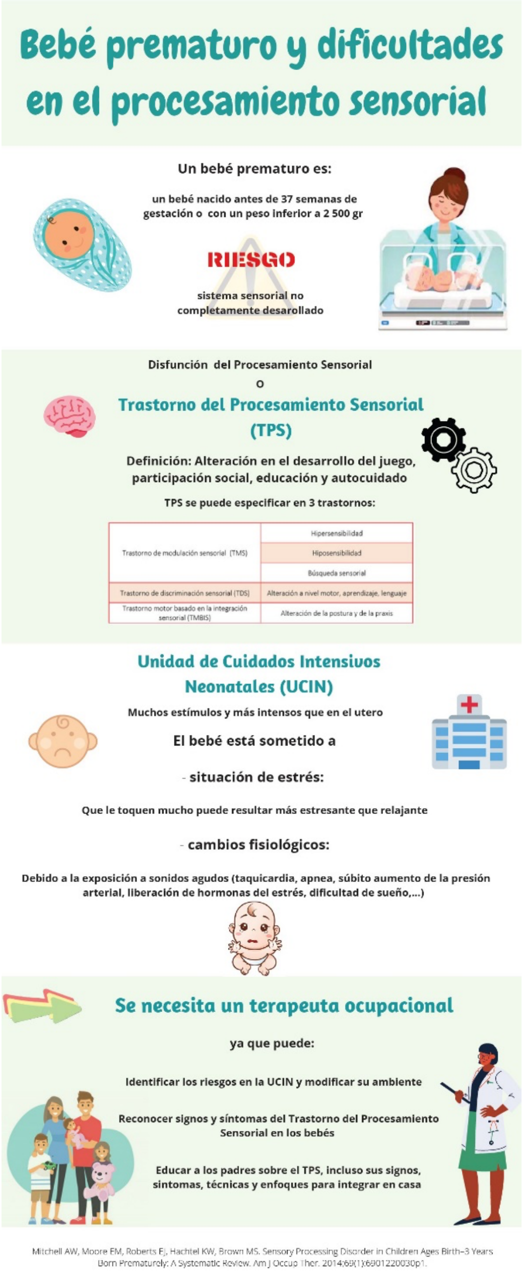

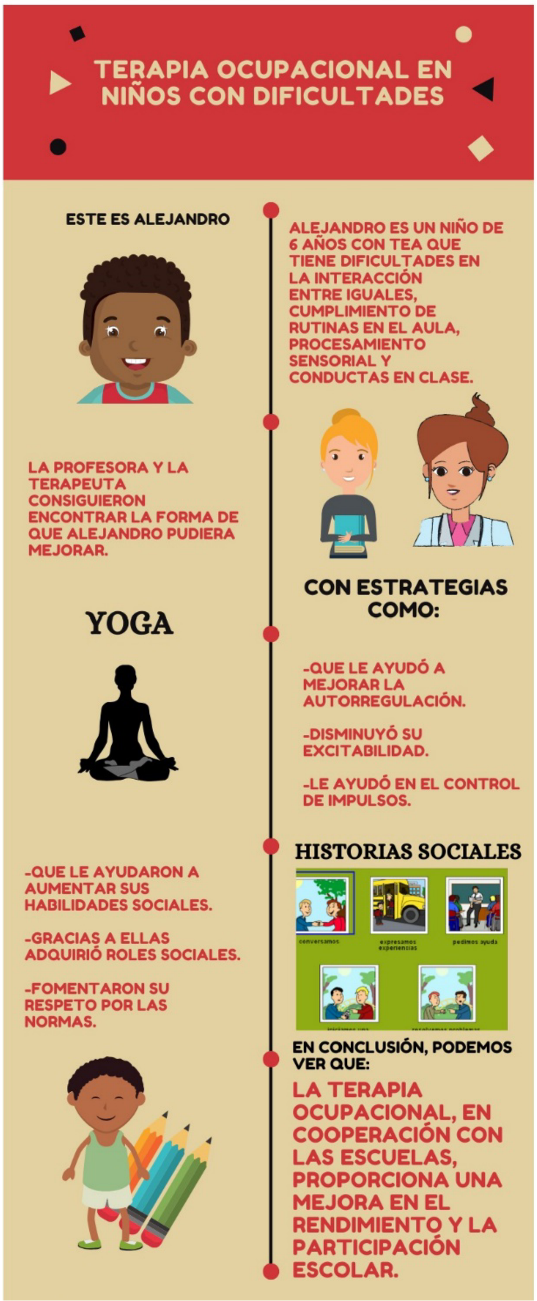

Examples of Podiatry students.

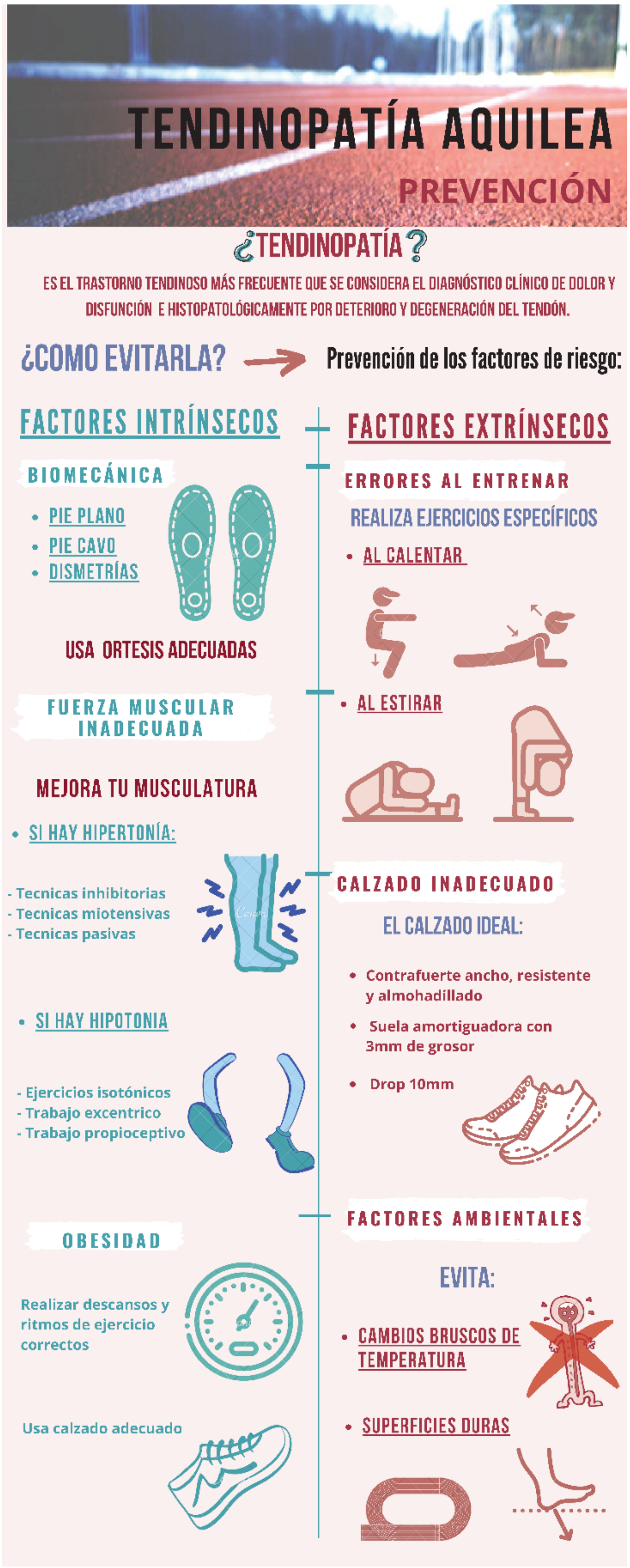

Supplement: Supplementary file 1 [file ejihpe-14-00060-s001.zip › ejihpe-2893591-supplementary.pdf]
